# Dr. Walter D. Greene

**Published:** 1917-09

**Authors:** 


**Dr. Walter D. Greene,** Buffalo, 1876, died of cerebral haemorrhage at West Falls, Aug. 3, aged 64. A full obituary notice by Dr. De Lancey Rochester will appear in our next issue.

